# Angiotensin I-Converting Enzyme (ACE) Inhibitory Activity and ACE Inhibitory Peptides of Salmon (*Salmo salar*) Protein Hydrolysates Obtained by Human and Porcine Gastrointestinal Enzymes

**DOI:** 10.3390/ijms150814077

**Published:** 2014-08-13

**Authors:** Małgorzata Darewicz, Justyna Borawska, Gerd E. Vegarud, Piotr Minkiewicz, Anna Iwaniak

**Affiliations:** 1 Department of Food Biochemistry, Faculty of Food Science, University of Warmia and Mazury in Olsztyn, Olsztyn 10-726, Poland; E-Mails: justyna.borawska@uwm.edu.pl (J.B.); minkiew@uwm.edu.pl (P.M.); ami@uwm.edu.pl (A.I.); 2 Department of Chemistry, Biotechnology and Food Science, Norwegian University of Life Sciences, Ås NO-1432, Norway; E-Mail: gerd.vegarud@nmbu.no

**Keywords:** human gastrointestinal enzymes, commercial porcine enzymes, salmon, ACE-inhibitory peptides

## Abstract

The objectives of the present study were two-fold: first, to detect whether salmon protein fractions possess angiotensin I-converting enzyme (ACE) inhibitory properties and whether salmon proteins can release ACE inhibitory peptides during a sequential *in vitro* hydrolysis (with commercial porcine enzymes) and *ex vivo* digestion (with human gastrointestinal enzymes). Secondly, to evaluate the ACE inhibitory activity of generated hydrolysates. A two-step *ex vivo* and *in vitro* model digestion was performed to simulate the human digestion process. Salmon proteins were degraded more efficiently by porcine enzymes than by human gastrointestinal juices and sarcoplasmic proteins were digested/hydrolyzed more easily than myofibrillar proteins. The *ex vivo* digested myofibrillar and sarcoplasmic duodenal samples showed IC_50_ values (concentration required to decrease the ACE activity by 50%) of 1.06 and 2.16 mg/mL, respectively. The *in vitro* hydrolyzed myofibrillar and sarcoplasmic samples showed IC_50_ values of 0.91 and 1.04 mg/mL, respectively. Based on the results of *in silico* studies, it was possible to identify 9 peptides of the *ex vivo* hydrolysates and 7 peptides of the *in vitro* hydrolysates of salmon proteins of 11 selected peptides. In both types of salmon hydrolysates, ACE-inhibitory peptides IW, IY, TVY and VW were identified. In the *in vitro* salmon protein hydrolysates an ACE-inhibitory peptides VPW and VY were also detected, while ACE-inhibitory peptides ALPHA, IVY and IWHHT were identified in the hydrolysates generated with *ex vivo* digestion. In our studies, we documented ACE inhibitory *in vitro* effects of salmon protein hydrolysates obtained by human and as well as porcine gastrointestinal enzymes.

## 1. Introduction

Bioactive peptides are inactive when they are encrypted in the parental protein but can be enzymatically released and reveal various biological activities. Bioactive peptides of food origin can act as biological regulators or neurotransmitters. They may express a variety of functions in the gastrointestinal tract or in the intestinal epithelium or after systemic absorption into blood circulation [1]. Angiotensin I-converting enzyme (ACE) (EC 3.4.15.1) is a hypertension-responsible glycoprotein present both in biological fluids and many tissues [2]. ACE inhibitors are the most-studied food peptides and display different biological functions [2,3]. Since fish proteins are structurally diversified, they may serve as a substrate to produce peptides with multifunctional bioactivities [4]. Fish protein hydrolysates have particularly interested food biotechnologists due to the availability of a highly-balanced amino acid content, a high nitrogen content and the presence of biologically active peptides [5,6]. Using fish as a source of bioactive peptides provides a novel tool for the introduction of potentially high-value bioactive products.

There are several published studies reporting the presence of bioactivities in enzymatically hydrolysed fish proteins, including ACE inhibitors/antihypertensive peptides [6]. The first report confirming the presence of peptides possessing ACE inhibitory activity in fish protein hydrolysates was published in 1986 for sardine and hairtail [7]. More recently, Gehring *et al.* [8] reported that fish protein hydrolysates have been shown to inhibit ACE. The peptide LKPNM with an ACE inhibitory activity was found in traditional Japanese food, called katsuobushi, a fermented, smoked skipjack tuna (*Katsuwonus pelamis)* [6,9]. Actin of skipjack tuna was reported to be a source of 8 peptides with confirmed ACE inhibitory activity [6,9]. Tuna dark muscle treated with various proteases such as alcalase, neutrase, pepsin, papain, α-chymotrypsin and trypsin also released ACE inhibitory peptides [10,11]. Six dipeptides recognized as ACE inhibitors/antihypertensive agents were identified in the thermolysin hydrolysate of upstream chum salmon [12]. The same authors [13] demonstrated the antihypertensive effect of salmon muscle hydrolysates on spontaneously hypertensive rats (SHR). Enari *et al.* [14] examined the antihypertensive effect of salmon hydrolysate containing 20 active di- and tripeptides. Peptides derived from Atlantic salmon skin were reported to possess potent ACE inhibitory properties [15]. Ewart *et al.* [16] used salmon rack proteins and hydrolysed them by alcalase, flavourzyme, fungal protease concentrate, Protease GC106, Multifect Neutral and Protease S-Amano to produce bioactive peptides. Several peptides were found in a salmon protein hydrolysate with sequences as follows: LAF, LTF, IIF, LAY, IAY, VFY, YAY, VLW, IAW, YAL, YNR. All of the peptides showed activity against the ACE.

Farmed salmon (*Salmo salar*) is a popular food around the world with high nutritional value and a potential application in the prevention of diet-related diseases. There is scientific evidence that diet has a direct relationship with cardiovascular diseases [17]. However, there is no published information available on the ability of salmon proteins to generate ACE inhibitory peptides during gastrointestinal digestion. In the present study, the main purpose was to detect whether salmon protein fractions contain amino acid sequences that have ACE inhibitory properties and whether salmon proteins can release ACE inhibitory peptides during the sequential *in vitro* hydrolysis (with commercial porcine enzymes) or *ex vivo* digestion (with human gastrointestinal enzymes). ACE inhibitory activity was also assayed for salmon protein hydrolysates.

## 2. Results and Discussion

This study was divided into three following parts: *in silico*, *ex vivo* and *in vitro*. In the *in silico* stage we used the information annotated in BIOPEP database to determine the potential biological activity profile to find ACE inhibitory peptides encrypted in selected salmon proteins. Since the presence of “hidden” peptides does not indicate a possible release of those biopeptides, we used computer software to simulate proteolysis to find ACE inhibitory peptides that can potentially be generated according to the specificities of pepsin, trypsin and chymotrypsin. Based on the *in silico* results, fragments with ACE inhibitory activity were selected and were further identified in the hydrolysates of myofibrillar and sarcoplasmic salmon proteins (Table 1).

**Table 1:** Amino acid sequences of ACE-inhibitory peptides in the “gastric” and “duodenal” hydrolysates of myofibrillar (M) and sarcoplasmic (S) salmon proteins, IC_50_ (concentration required to decrease the ACE activity by 50%) value and hydrophobicity.

Sequences of ACE Inhibitors	Type of Salmon Proteins	IC_50_ (μM)	Hydrophobicity (Kyte-Doolittle Scale)
IVY	M, S	0.48	7.4
VW	M, S	1.4	3.3
IY	M, S	2.1	3.2
IW	M, S	4.7	3.6
VY	M, S	7.1	2.9
TVY	M	15	2.2
VFPS	M	0.46	4.6
VTVNPYKWLP	M	5.5	−1.3
IWHHT	M	5.8	−3.5
YALPHA	M	9.8	1.3
ALPHA	M	10	2.6

ACE inhibitory activity, expressed as a value of IC_50_, as an indicator of an enzyme’s biological response to a dose of a biopeptide, was a criterion for selecting peptides used in further stages of the studies in order to identify them with mass spectrometry. During *in vitro* studies of peptides with the sequences: TVY, VFPS, VTVNPYKLWLP, YALPHA and ALPHA, they were recognized as ACE inhibitors [18,19,20,21,22,23]. Peptides with the sequences: IVY, VW, IY, IW, VY and IWHHT were experimentally recognized as antihypertensive *i.e.*, lowering blood pressure in SHR after oral administration [24,25,26,27]. The value of hydrophobicity, *i.e.*, one of the physicochemical properties of the examined peptides, was used; this parameter is important during separation assays with RP–HPLC [28] and for the course of ACE inhibition properties [2]. These results are consistent with the results reported by others where ACE peptide inhibitors derived mainly from myofibrillar proteins and collagen were identified [14,15]. The presented strategy mimics the so-called “hypothesis-driven proteomics” [29] and it may be named, by analogy, “hypothesis-driven peptidomics”. This strategy consists in selecting peptides based on the results of bioinformatics studies with further identification. In the case of biologically active peptides, it is possible to search for fragments that are identical with previously-collected sequences stored in databases [30] or to predict the activity of protein fragments with such software applications as PeptideRanker [31].

The bioinformatics approach presented above was recently successfully applied to study the distribution of ACE-inhibiting peptides within their primary structure of typical food proteins, e.g., from peanuts, amaranthus, whey, champignon mushroom *Agaricus bisporus* [32,33,34,35,36].

However, it should be noted that the results generated by the *in silico* simulation of proteolysis were not necessarily confirmed in *in vitro* studies due to fundamental simplification assumed for the availability of all bonds susceptible to a given enzyme in the polypeptide chain of an examined protein or incomplete data on the specificity of an enzyme. The results of the studies by Thewissen *et al.* [37] are an example of the failure to implement such a strategy. The experimental data collected during that study did not confirm the theoretical prognoses of the potential to release ACE inhibitors by prolyl endopeptidase (EC 3.4.21.26**)**. The specificity of the enzymes is probably narrower than that described in different repositories, including BIOPEP. The accumulation of as many peptide sequences as possible in databases and their constant updating as well as replenishing information on the specificity of enzymes used in *in silico* hydrolysis are a factor that determines the level of efficacy and the potential to release biopeptides. The efficacy and verifiability of results generated with *in silico* assays for predicting the potential to release bioactive peptides from food proteins were discussed in a paper by Vercruysse *et al.* [38].

### 2.1. Protein Content and Sodium Dodecyl Sulfate Polyacrylamide Gel Electrophoresis Analysis of ex Vivo and in Vitro Salmon Protein Hydrolysates

**Figure 1:**
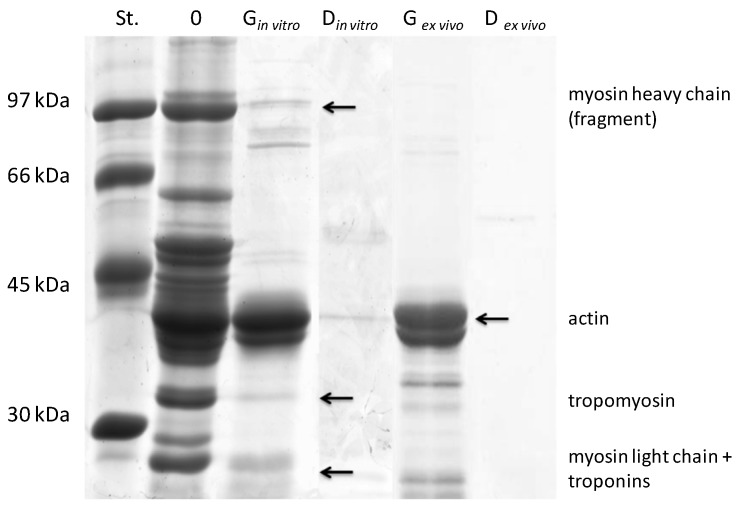
Electrophoretic separation (SDS-PAGE) of the hydrolysates of salmon myofibrillar proteins after *ex vivo* digestion (*ex vivo*) or *in vitro* hydrolysis (*in vitro*). Standard (St.)-mass marker (97–30 kDa), G-samples after the 2 h “gastric”/pepsin phase, D-samples after 1 h “duodenal”/pepsin + Corolase PP phase, “0”-samples after the “chewing” phase (see the “Methods” section).

**Figure 2:**
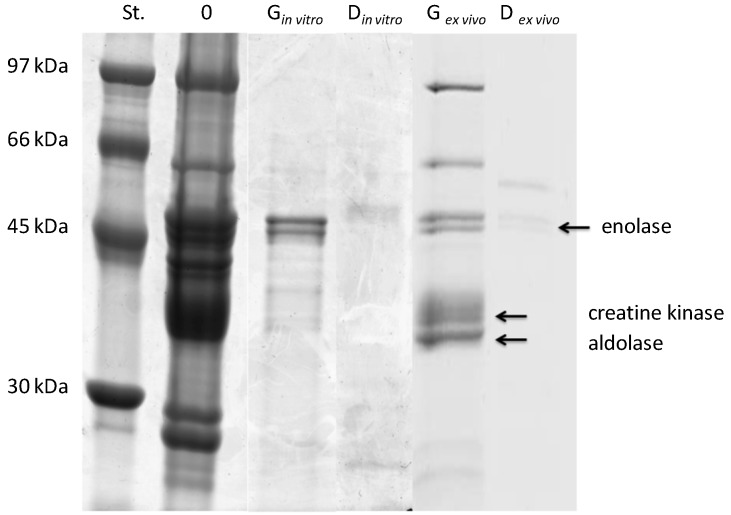
Electrophoretic separation (SDS-PAGE) of the hydrolysates of salmon sarcoplasmic proteins after *ex vivo* digestion (*ex vivo*) or *in vitro* hydrolysis (*in vitro*). Standard (St.)-mass marker (97–30 kDa), G-samples after the 2 h “gastric”/pepsin phase, D-samples after 1 h “duodenal”/pepsin + Corolase PP phase, “0”-samples after the “chewing” phase (see the Methods Section).

Figure 1 and Figure 2 show the SDS-PAGE protein profiles of non-digested/non-hydrolyzed and *ex vivo* digested/*in vitro* hydrolyzed myofibrillar and sarcoplasmic proteins of salmon, respectively. The results indicated that the protein bands of non-digested/non-hydrolyzed samples have molecular weights ranging from ~20 to 97 kDa and above. The highest molecular weight of protein bands, *i.e.*, ~97 kDa and above, are likely to represent the subunits of myosin as reported by Ojagh *et al.* [39]. The protein bands with a molecular weight of 45 kDa are considered as the actin fraction and three lower molecular weight bands (~30, ~20 and ~14 kDa) may correspond to the tropomyosin, myosin light chain and globin fractions. These results are in line with the results of the studies by Martinez [40] and Lin [41].

In the gastric or pepsin phase, the highest molecular weight proteins bands (<45 kDa) were significantly hydrolyzed into smaller peptides. The intensity of these protein bands was highly reduced in all hydrolysates. The additional new bands were observed for myofibrillar protein patterns of ~35 kDa and less. It is interesting to note that the protein bands corresponding to the apparent molecular mass of actin remained nearly intact for all hydrolysates, suggesting that this subunit is rather stable in the part of the protein and hindered access from gastric juices and pepsin. Similar relations were reported by Eriksen *et al.* [42] who compared the course of *in vitro* hydrolysis of milk proteins with pepsin with hydrolysis with human gastric juices.

Duodenal or pepsin A and Corolase PP phases had a significant effect on the proteolysis of both myofibrillar and sarcopasmic salmon proteins. In the presence of duodenal juice and/or Corolase PP, it was observed that the enzymatic hydrolysis led to a gradual breakdown of proteins. Figure 1 and Figure 2 show that the majority of proteins were hydrolysed after the duodenal phase or the pepsin and Corolase PP phase. For instance, Inglingstad *et al.* [43] observed an almost immediate hydrolysis of casein from sheep, goat and equine milk within 5 minutes of *in vitro* hydrolysis with human digestive juices.

Similar trends were reported in other studies for hydrolysates prepared from goat milk by Eriksen *et al.* [44] who indicated that the rate of “duodenal” digestion may be impacted by bile salts from human digestion juices, which are a complex mixture of bile salts and proteases, amylases and lipases in different isoforms in combination with inhibitors, activators, bilirubin and other compounds which may influence the hydrolysis of proteins.

The observed differences are in line with the results presented in Table 2, which shows the percentage of protein remaining intact after *ex vivo* digestion and *in vitro* hydrolysis of salmon proteins.

**Table 2:** The percentage of protein remaining intact after *ex vivo* digestion and *in vitro* hydrolysis of salmon proteins.

Hydrolysates	Myofibrillar Proteins		Sarcoplasmic Proteins	
	G/Pepsin A	D/Pepsin A and CPP	G/Pepsin A	D/Pepsin A and CPP
***Ex vivo* digestion**	41.03 ± 1.77	9.82 ± 1.54	20.06 ± 0.28	1.09 ± 0.00
***In vitro* hydrolysis**	27.73 ± 2.70	2.49 ± 0.85	5.23 ± 1.17	0.44 ± 0.14

G, gastric phase with human gastric juice; D, duodenal phase with human duodenal juice following gastric juice; and CPP, Corolase PP. Data are presented as the means ± SD. The values in the same row for myofibrillar and sarcoplasmic proteins differ significantly at *p* ≤ 0.05. The values in the same column presented separately for myofibrillar and sarcoplasmic gastric/pepsin proteins differ statistically significantly at *p* ≤ 0.05.

Myofibrillar proteins were more resistant to digestion or hydrolysis at the two studied stages compared to sarcoplasmic proteins. Such behavior of myofibrillar proteins during depolymerisation is explained by the spatial structure of actomyosin and the presence of lipids which usually hinder the activity of proteolytic enzymes [45].

Based on the amount of intact myofibrillar and sarcoplasmic proteins remaining, porcine pepsin and pepsin followed by Corolase PP were more efficient than human gastric and duodenal juices, respectively. Moreover, the greatest degradation, leaving nearly no intact protein, was observed for hydrolysate of sarcoplasmic salmon proteins after using pepsin A and Corolase PP.

### 2.2. Angiotensin I-Converting Enzyme Inhibitory Activity of Hydrolysates

**Figure 3:**
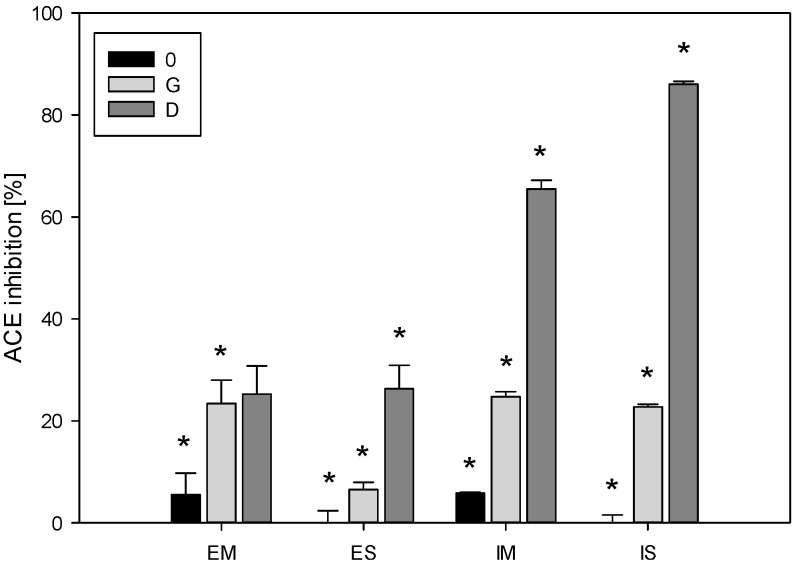
Angiotensin I-converting enzyme (ACE) inhibitory activities (expressed as % ACE-inhibitory activity of digested (E) and hydrolysed (I) myofibrillar (M) and sarcoplasmic (S) proteins from salmon. 0-a sample after the “chewing” stage, G-a sample after the 2 h “gastric” stage, D-samples after the 1 h “duodenal” stage. Value means ± SD. of three determinations. The pillars denoted with “*” differ statistically at *p* < 0.05.

Salmon myofibrillar and sarcoplasmic proteins were digested using human gastrointestinal juices or hydrolysed using pepsin and Corolase PP. ACE inhibitory activities of hydrolysates are shown in Figure 3. Figure 3 shows that *ex vivo* hydrolysate from salmon myofibrillar proteins had the ability to inhibit the ACE in the range of 6% to 25%, whereas the *in vitro* hydrolysate was in the range of 6% to 66%. In turn, *ex vivo* hydrolysate from salmon sarcoplasmic proteins had the ability to inhibit the ACE in the range of 0% to 26%, whereas the *in vitro* hydrolysate was in the range of 0% to 86%. When the ACE inhibitory activity is expressed as the concentration required to inhibit 50% of ACE activity (IC_50_), *ex vivo* digested myofibrillar and sarcoplasmic duodenal samples showed IC_50_ values of 1.06 and 2.16 mg/mL, respectively. *In vitro* hydrolyzed myofibrillar and sarcoplasmic pepsin + Corolase PP samples showed IC_50_ values of 0.91 and 1.04 mg/mL, respectively. These results correspond with the results shown in the section: “Protein content and SDS-PAGE analysis of *ex vivo* and *in vitro* salmon protein hydrolysates”. An increase in the ACE inhibitory activity during a time extension of hydrolysis was also recorded for proteins in Atlantic salmon hydrolyzed with Alcalase and papain [15]. In our study, 60-min salmon duodenal hydrolysates showed a slightly decreased ACE inhibition than that reported by Nakajima *et al.* [46] when the pepsin and pancreatin hydrolysates of Atlantic salmon showed a IC_50_ value of 0.791 mg/mL. Extracts from pickled mackerel, fermented mackerel, sardine muscle hydrolysate and hard clam extract were reported to have IC_50_ values of 0.1–0.4, 0.06–0.20, 0.25–0.62 and 0.036–1.090 mg/mL, respectively [46]. Most of the inhibitory peptides derived from marine proteins were reported to be short-chain and were obtained from hydrolysates with a high degradation level [2]. The results presented here suggest that ACE inhibitory peptides can be produced with human gastrointestinal juices as well as commercial porcine enzymes. The ACE-inhibitory activities of digested/hydrolyzed samples increased with the time applied. The gastric stage of digestion followed by a duodenal stage and pepsin stage of hydrolysis followed by Corolase PP stage seem to be the optimal scheme to produce peptides with a high potency of ACE-inhibitory activity. Smaller peptides produced in these final stages of digestion/hydrolysis are known to be more potent ACE inhibitors than larger peptides, likely because they better fit into an ACE active site aimed to change its activity [2]. Furthermore, the results showed significant differences between peptide patterns obtained via *ex vivo* digestion and *in vitro* hydrolysis. Human gastrointestinal enzymes generated peptides with weaker ACE inhibitory activity of the hydrolysates than the activity of peptides from hydrolysates obtained with commercial porcine enzymes. These results are in line with the results reported by Eriksen *et al.* [44] who concluded that commercial enzymes appeared to digest whey proteins more efficiently than human digestive juices.

### 2.3. Identification of Angiotensin I-Converting Enzyme (ACE) Inhibitory Peptides

The “gastric-duodenal” *ex vivo* and *in vitro* hydrolysates of myofibrillar and sarcoplasmic salmon proteins were separated with reversed-phase high performance liquid chromatography coupled with mass spectrometry (RP-HPLC–MS/MS). The ACE inhibitory fragments selected based on the results of *in silico* studies were identified in the hydrolysates of myofibrillar and sarcoplasmic salmon proteins. The analysis of chromatograms generated for the mass-to-charge ratio (*m*/*z*) of many fragment ions derived from a selected precursor ion allowed the experimental retention time for peptides to be determined. The peptides with four amino acid residues or more were regarded as identified if the experimental and predicted retention times did not differ by more than 10% [28]. Di- and tripeptides for which large differences were found between predicted and experimental retention time were regarded as identified if the experimental retention times in the tested samples were comparable (±1 min) to the predicted values.

**Figure 4:**
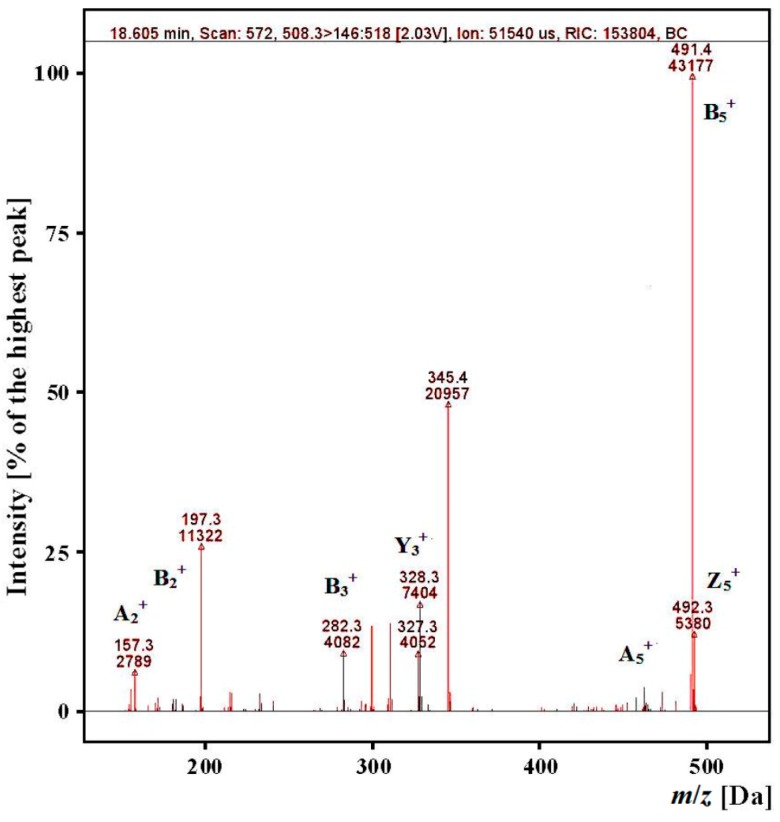
Mass spectra of fragment ions derived from the precursor ion with *m*/*z* = 508.3 from the “gastric-duodenal” hydrolysate produced with *ex vivo* digestion of myofibrillar salmon proteins (*t*_R_ app. 18 min) obtained during identification of ALPHA-sequence peptide.

**Figure 5:**
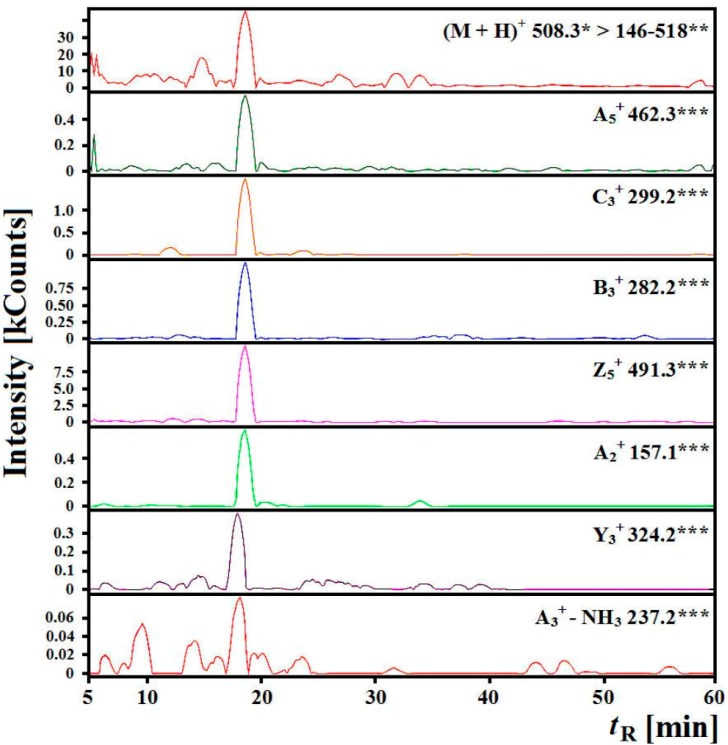
Chromatograms of fragment ions derived from the precursor ions with *m*/*z* = 508.3 from the “gastric-duodenal” hydrolysate generated with *ex vivo* digestion of myofibrillar salmon proteins obtained during identification of ALPHA sequence peptide, where * *m*/*z* for the precursor ion, ** range of *m*/*z* for investigated fragment ions, *** a type of ion and *m*/*z* of the fragment ion, kCounts—count × 10^3^, *t*_R_ predicted = 17.57 min, *t*_R_ experimental = 18.59 min.

For example, it was possible to identify a peptide with ALPHA amino acid sequence and *m*/*z* = 508.3 in the gastric-duodenal *ex vivo* hydrolysate because of the presence of *m*/*z* values for fragment ions (determined by use of the calculator for fragment ions in peptides) in the analyzed spectra (Figure 4) and the presence of fragment ions in the chromatograms (Figure 5) for which the experimental retention time of 18.59 minutes was comparable (±10%) to the predicted time of 17.57. The *m*/*z* value, the type of detected fragment ion [47] and a released neutral particle (ammonium) are highlighted on the chromatograms.

The analysis of chromatograms and mass spectra for investigated *m*/*z* values of precursor ions and fragment ions led to determine the experimental retention times for all peptides selected according to the procedure described in the section “Methods” (Table 3). During identification, fragment ions of A, B, Y and Z types were most commonly observed. The experimental retention time was not established if different retention times were detected on the chromatograms for fragment ions or no retention times were recorded (e.g., for the LEQQVDDLEGSLEQEKK peptide). Based on the above-mentioned criteria, it was possible to identify 9 peptides from the *ex vivo* hydrolysates of myofibrillar and sarcoplasmic salmon proteins of 11 peptides selected at the *in silico* stage.

**Table 3:** Experimental and predicted retention times (*t*_R_) for ACE-inhibitory peptides (ACEi) that were searched for in the “gastric-duodenal” hydrolysates obtained after *ex vivo* digestion of myofibrillar (M) and sarcoplasmic (S) salmon proteins; nd, fragment ions were not detected in a specific time period; “–”, peptides not analyzed because they are not present in the myofibrillar/sarcoplasmic protein sequences.

Sequences	*m*/*z*	*t*_R_ Predicted (min) **	*t*_R_ Experimental (min)	
			M	S
ALPHA	508.3	17.57 ± 1.76	18.59 *	–
IVY	394.2	–	–	33.11 *
IW	318.2	–	30.82 *	30.83 *
IWHHT	693.3	23.58 ± 2.36	25.10 *	–
IY	295.2	–	9.51 *	9.12 *
TVY	382.2	–	27.08 *	–
VFPS	449.2	24.50 ± 2.45	nd	–
VTVNPYKWLP	608.8 (2+)	36.69 ± 3.68	nd	–
VW	304.2	–	21.61 *	nd
VY	281.2	–	nd	34.00
YALPHA	671.4	24.04 ± 2.40	26.98	–

* experimental retention times that allowed the identification of a peptide; and ** prediction of retention time included possible error ±10% (see the “Methods” section); and (2+) doubly protonated peptide.

**Figure 6:**
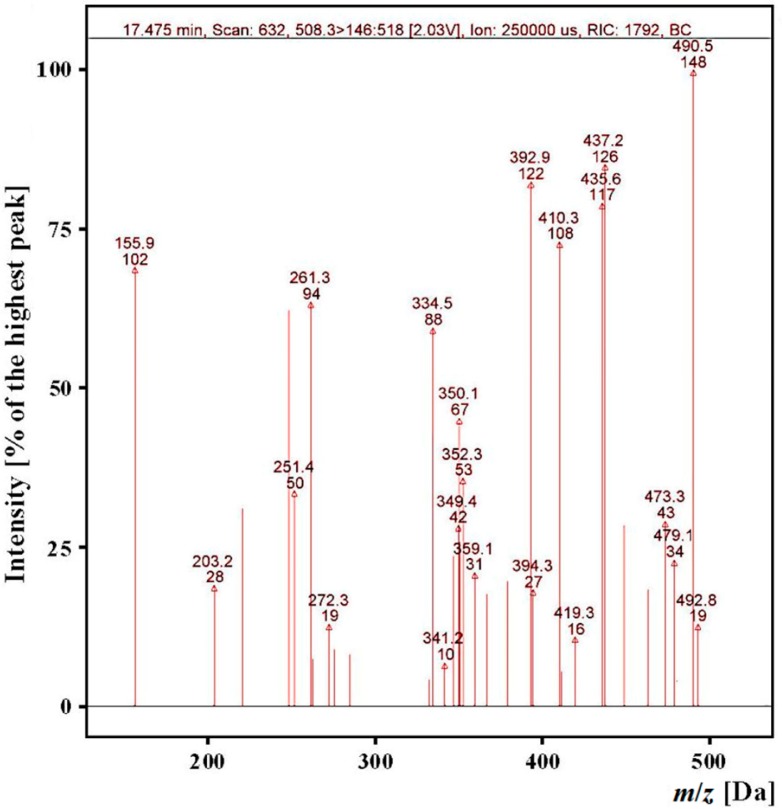
Mass spectra for fragment ions derived from the precursor ion with *m*/*z* = 508.3 from the *in vitro* hydrolysate of myofibrillar salmon proteins (A) after exposure to pepsin and Corolase PP (retention time: app. 18 min) obtained during identification of ALPHA sequence peptide.

**Figure 7:**
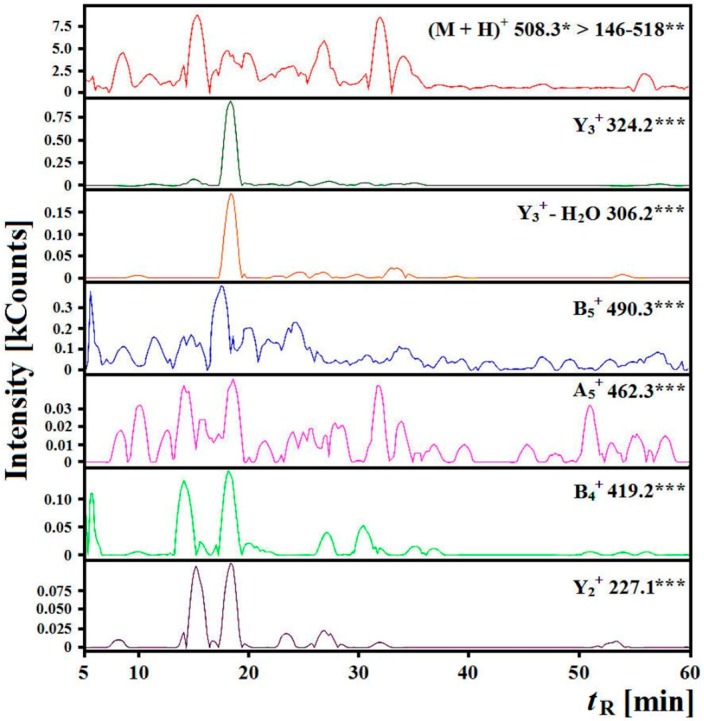
Chromatograms of fragment ions derived from the precursor ion with *m*/*z* = 508.3 from the *in vitro* hydrolysate of myofibrillar salmon proteins after exposure to pepsin and Corolase PP obtained during identification of ALPHA sequence peptide, where * *m*/*z* for the precursor ion, ** range of *m*/*z* for investigated fragment ions, *** a type of ion and *m*/*z* of the fragment ion, Counts, kCounts—count × 10^3^, *t*_R_ predicted = 17.57 min.

The *in vitro* hydrolysates of myofibrillar and sarcoplasmic salmon proteins were also separated with reversed-phase high performance liquid chromatography coupled with mass spectrometry (RP–HPLC–MS). The identification of an ALPHA peptide in the hydrolysate of myofibrillar salmon proteins was not possible because fragment ions appeared in several different time points whereas at the time that approximated the predicted value, *i.e.*, 18.32 min, few fragment ions flew out, which is further confirmed by the low number of counts at this time point (Figure 6 and Figure 7).

The analysis of chromatograms and mass spectra for demanded values of *m*/*z* ratio for the precursor ions and fragment ions allowed the experimental retention times for the majority of the investigated peptides (Table 4) to be determined. Similar to the identification of peptides in the hydrolysates obtained with the use of human digestive juices, fragment ions of A, B, Y and Z types were most commonly detected [47]. The retention time was not determined if different retention times were present on the chromatograms for fragment ions or retention times were not detected (e.g., for the above-mentioned ALPHA peptide).

**Table 4:** Experimental and predicted retention times (*t*_R_) for ACE-inhibitory peptides (ACEi) that were searched for in the hydrolysates generated after *in vitro* hydrolysis with pepsin and Corolase PP of myofibrillar (M) and sarcoplasmic (S) salmon proteins, nd, fragment ions were not detected in a specific time period; “–”, peptides not analyzed because they are not present in the myofibrillar/sarcoplasmic protein sequences.

Sequences	*m*/*z*	*t*_R_ Predicted (min) **	*t*_R_ Experimental (min)	
			M	S
ALPHA	508.3	17.57 ± 1.76	nd	–
IVY	394.2	–	–	nd
IW	318.2	–	29.58 *	31.05 *
IWHHT	693.3	23.58 ± 2.36	15.79	–
IY	295.2	–	6.61	8.29 *
TVY	382.2	–	27.26 *	–
VFPS	449.2	24.50 ± 2.45	nd	–
VPW	401.2	–	6.15 *	–
VTVNPYKWLP	608.8 (2+)	36.69 ± 3.68	43.15	–
VW	304.2	–	20.79 *	23.46
VY	281.2	–	nd	15.13 *
YALPHA	671.4	24.04 ± 2.40	27.88	–

* Experimental retention times that allowed the identification of a peptide; and ** Prediction of retention time included possible error ±10% (see the “Methods” section); (2+) doubly protonated peptide.

Based on the assumed criteria, it was possible to identify 7 peptides from the *in vitro* hydrolysates of myofibrillar and sarcoplasmic salmon proteins of 11 peptides selected at the *in silico* stage.

The analysis of the identification results carried out with LC-MS/MS indicate that in both types of salmon hydrolysates, ACE-inhibitory peptides with IW, IY, TVY and VW sequences were identified. In the hydrolysates of salmon proteins obtained with *in vitro* hydrolysis, an ACE-inhibitory peptides with VPW and VY sequence was additionally detected, whereas ACE-inhibitory peptides with ALPHA, IVY and IWHHT sequences were identified in the hydrolysates produced with *ex vivo* digestion.

Some of identified sequences were found in other food protein sources and showed ACE inhibitory/antihypertensive effect [2]. For example, the sequences IW, VW and VY were identified in wakame (*Undaria pinnatifida*). A dose of 1 mg of each peptide/kg body weight reduced systolic blood pressure (SBP) in SHR after 3 h following the administration [25]. Peptides ALPHA and IWHHT were derived from dried bonito treated with thermolysin. These sequences were found to inhibit ACE at the level shown in Table 1 [23]. The sequence IWHHT reduced the systolic blood pressure in SHR at the level of 60 mmHg (dose: 10 mg/kg body weight) [24]. Tripeptide IVY was found in a wheat germ hydrolysate and the antihypertensive effect of this sequence was tested on mice. An injection of IVY (dose: 5 mg/kg body weight) caused the maximum arterial blood pressure reduction (19.2 mmHg) after 8 min and the blood pressure reduction was held for 20 min. Further metabolism of IVY led to obtain ACE-inhibitory dipeptide VY [48]. Therefore, Matsui *et al.* [48] found the peptide IVY to be a blood pressure reductant due to its antihypertensive effect and the release of VY dipeptide after IVY absorption. The sequences IVY and VY were also derived from Royal Jelly (*Apis mellifera*), a food regarded as beneficial for health. A single oral administration of VY and IVY (1 and 10 mg/kg body weight, respectively) significantly reduced the systolic blood pressure in rats within one hour after administration. The reducing effect of these two peptides lasted for 8 h [27].

The peptides were identified based on an analysis of fragment ions using a mass-to-charge ratio calculated with bioinformatics tools. The identification of peptides composed of at least 4 amino acids was also based on a comparison between theoretical and experimental retention times. The equation described in the section “Methods” that was used to calculate the theoretical retention times for peptides differed from the one included in the cited reference [28]. The reason for these differences was a modification in the SSRCalc software introduced after the paper by Dziuba *et al.* [28] was released. 

During identification of some peptides, different retention times for fragment ions (*i.e.*, opposite to co-elution, which is the impact of different peptides with the same retention time) and the relatively few counts compared to the precursor ion were observed. The above-mentioned ions may originate from the following categories of peptides which were specified by Alves *et al.* [49]: (1) peptides which are isobaric to each other (with the same molecular weight) and cannot be separated by a mass spectrometer based on their mass; (2) peptides with comparable molecular masses that cannot be separated by a spectrometer due to limited apparatus resolution; and (3) peptides with different charges yet with the same or approximate *m*/*z* ratio (within the resolution range of an apparatus) such as fragment ions typical of the sought compounds.

The identification of peptides with LC-MS/MS based on the detection of fragment ions and a comparison of their retention times is hindered, among others, by the occurrence of non-sequential ions or the regrouping of ions that are missed during analyses with the ion trap [50]. Moreover, in the case of ESI-type ionization that was applied in the present studies, the potential occurrence of ions depends on such parameters as the concentration of an analyzed sample, the flow velocity of a dissolvent or the difference in potentials (*i.e.*, the difference between the voltage of a needle and a shield) [51].

The potential to detect peptides based on theoretical predictions has been discussed [1,3,52,53]. The theoretical potential for using such a strategy was presented by Minkiewicz *et al.* [3]. The studies involved both the identification of biologically active peptides [1,53] and the application of peptides as markers of allergenic proteins [52].

Although the published results of *in silico* analyses usually cover successful prognoses [54], there are examples in the literature of discrepancies between the theoretical predictions and observed biological activity of protein hydrolysates [37,55]. Tulipano *et al.* [55] found a peptide that inhibited dipeptidyl peptidase IV (EC 3.4.14.5) in a whey protein hydrolysate; this peptide was not detected with the profile of the potential activity of any of the examined proteins. Thewissen *et al.* [37] reported that ACE-inhibitory activity determined for the products of wheat protein hydrolysis with proline endopeptidase (EC 3.4.21.26) was lower than the analogous activity for the products of hydrolysis of the same proteins with thermolysin (EC 3.4.21.64) even though the *in silico* prediction suggested a different result. Potential reasons behind these discrepancies were discussed by Minkiewicz *et al.* [53]. The cause of these discrepancies could be the presence of peptides that were not recorded in databases and differences between the predicted and actual specificity of enzymes.

The detection of peptides with the strategy applied in the present study requires proteolytic enzymes to release a sought fragment of the protein chain without hydrolysing the bond inside a given peptide. In addition, this fragment should not undergo chemical or enzymatic modifications. Such modifications may prevent hydrolysis of peptide bonds as well as change the mass of a peptide and fragment ions. Amino acid residues of peptides and proteins may undergo numerous chemical modifications. For instance, the modification of base residues (of lysine and arginine) prevents hydrolysis of the protein chain by trypsin. Modifications of other amino acid residues change the molecular weight of protein fragments, which may prevent their identification with MS and MS/MS [56].

According to Mallick *et al.* [57], each type of a mass spectrometer detects a different set of peptides. Darewicz *et al.* [58] reported a case for which the presence of peptides with aromatic amino acids in the hydrolysates of bovine β-casein was confirmed based on UV spectra even though the analysis with MALDI-ToF-MS did not demonstrate such peptides. Although the potential to detect individual peptides by a specific type of apparatus may be predicted based on the physicochemical properties of amino acid residues, a successful prediction requires analyzing at least hundreds of peptides [57].

Peptide VTVNPYKWLP contains bonds theoretically susceptible to trypsin, chymotrypsin and pepsin action as pointed out by Udenigwe and Howard [59]. Its presence in hydrolysate obtained *in vitro* indicates that some of the above-mentioned bonds were resistant to the proteolysis or only partially hydrolyzed in the conditions applied in this experiment. Absence of the peptide in *ex vivo* hydrolysate of myofibrillar proteins is in agreement with suggestions of Udenigwe and Howard [59] that joint action of proteolytic enzymes from the human gastrointestinal juices should lead to degradation of this fragment. Peptide VTVNPYKWLP could not be expected in the hydrolysate of sarcoplasmic proteins due to the fact that it is a myosin fragment.

The comparison between the results of peptide identification in the hydrolysates of salmon proteins obtained with human gastrointestinal juices and the results recorded after the application of enzymatic preparations derived from the porcine pancreas suggests that different peptides may be released from the same protein. The differences may be caused by spontaneous modifications of amino acid residues. However, the difference in the specificity of the applied enzymes is the most probable rationale. Among pancreatic serine proteases, high interspecies diversity (structural and functional) and a variety of forms (catalytic, isoenzymes) within a species has been observed [60]. For instance, trypsin in humans is synthesized in three molecular forms whereas there are two forms in the pig. It cannot be excluded that the applied duodenal juice isolated from volunteers might contain enzymes originating from microbiota in the digestive tract. Considering the complexity of digestion, the results obtained for *in vivo* digestion or, as in the present study, for *ex vivo* digestion, may differ from *in vitro* laboratory hydrolysis [61].

## 3. Experimental Section

### 3.1. Materials

Fresh salmon (*Salmo salar*) fillets were purchased directly from a local market (Olsztyn, Poland) and transported immediately to the laboratory on ice. Fish samples were averaged from fragmented 2 kg of skinless fillets, washed, packed and stored in a freezer at −70 °C. Pepsin, sodium dodecyl sulphate (SDS), ACE enzyme extract from rabbit lung, hippuryl-l-histidyl-l-leucine (HHL) and phosphate buffered saline (PBS), pyridine, benzene sulphonyl chloride (BSC) were purchased from Sigma-Aldrich (St. Louis, MO, USA), while LMW-SDS Calibration kit was supplied by GE Healthcare Life Sciences (Little Chalfont, Buckinghamshire, UK), Corolase PP was purchased from AB Enzymes (Darmstadt, Germany) and Protein Assay Dye Reagent Concentrate and bovine serum albumin (BSA) were obtained from Bio-Rad (Hercules, CA, USA). All other chemicals used in the experiments were of analytical grade. Highly purified water was prepared with Milli-Q PLUS (Millipore Corp., Bedford, MA, USA) and used for the preparation of all buffers and solutions.

The amino acid sequences of proteins from salmon (*Salmo salar*) were taken from the UniProt database available at the website http://www.uniprot.org (accessed between March and June 2012) [62]. Actin and myosin from the myofibrillar fraction, parvalbumin and myoglobin from the sarcoplasmic fraction, hemoglobin, heat shock protein, serum albumin and collagen from the other fractions were taken into consideration. In the UniProt database, there were up to several dozen amino acid sequences of certain proteins with the same names and functions. In order to reject identical sequences, ClustalW2-Multiple Sequence Alignment software (http://www.ebi.ac.uk/Tools/msa/clustalw2/) with standard settings was applied [63,64]. Amino acid sequences for which the degree of identity was less than 90% were selected for further studies.

### 3.2. Methods

#### 3.2.1. *In Silico* Assay

The BIOPEP database (http://uwm.edu.pl/biochemia) was updated with sequences of ACE inhibitory peptides originating from food [65,66]. The profiles of the potential biological activity of 52 amino acid sequences of salmon proteins selected from the UniProt database were determined. The profiles of potential biological activity were used to define the type and location of ACE inhibitory fragments in the protein chains. Proteolysis simulation in order to assess the possibility of ACE inhibitory peptide release was performed using a procedure built into the BIOPEP database. Data for pepsin (EC 3.4.23.1), trypsin (EC 3.4.21.4) and chymotrypsin (EC 3.4.21.1) were used. The value of hydrophobicity for the obtained peptides was calculated as the sum of hydrophobicity values for individual amino acids contained in the peptides according to the scale by Kyte and Doolitle [67].

#### 3.2.2. Extraction of Myofibryllar Proteins from Salmon Muscle Tissue

Myofibrillar proteins were extracted from salmon muscles according to the method described by Martinez *et al.* [40] with some modification. Cooled and comminuted muscle tissue (3 g) and 40 mL of cooled solution A (40 mM TRIS, pH 10.5) were homogenized in a Waring 8011E blender (Waring, Torrington, CT, USA) and then centrifuged for 5 min at 4 °C and approx. 20,000× *g* (Sigma 3K30 laboratory centrifuge, Sigma Laborzentrifugen GmbH, Osterode, Germany). The supernatant was immediately collected and stored at −70 °C. Extraction was performed in 3 repetitions which, after mixing and freeze drying, were used as the material for further studies.

#### 3.2.3. Extraction of Sarcoplasmic Proteins from Salmon Muscle Tissue

Sarcoplasmic proteins were extracted from salmon muscle tissue according to the method described by Carrera *et al.* [68] with some modification. 200 mL of 10 mM Tris-HCl buffer (pH 7.2) with 5 mM PMSF (phenylmethanesulphonyl fluoride) was added to the fish muscle tissue. The sample was then homogenized in a Waring 8011 blender and centrifuged for 20 min at 4 °C and 40,000× *g* (Sigma 3K30 laboratory centrifuge, Osterode, Germany). The supernatant was then filtered (nylon filters, NL16, 0.22 μm, 47 mm, Whatman^®^, Maidstone, UK) and the filtrate was frozen at −70 °C. Extraction was done in 3 repetitions. The samples from the repetitions after mixing and freeze-drying were used as the material for further studies.

#### 3.2.4. *Ex Vivo* Digestion

The *ex vivo* digestion method was performed according to Almaas *et al.* [69,70] with some modifications. Human gastric juice (HGJ) and duodenal juice (HDJ) were collected according to Ulleberg *et al.* [71]. All gastric and duodenal enzymes used in the study were obtained from six healthy adults.

Proteolytic enzyme activity was measured according to methods described by Ulleberg *et al.* [71]. HGJ was analyzed for pepsin activity at pH 3.0 with hemoglobin (Sigma, St. Louis, MO, USA) as substrate according to Sanchez-Chiang *et al.* [72]. HDJ was analyzed for total proteolytic activity at pH 8.0 with casein (Merck Co., Darmstadt, Germany) as substrate, as described by Krogdahl and Holm [73] and Kirschenbaum [74]. Briefly, it was done as follows: three concentrations of human gastric or duodenal juices were incubated with substrate for 10 min at 37 °C, and the reactions were stopped by the addition of trichloroacetic acid. After an overnight sedimentation at 4 °C, the samples were centrifuged for 10 min at 3000× *g*. Analyses were done in triplicates. One unit of enzyme activity was defined as the volume (mL) of gastric or duodenal juice giving a difference in absorbance of 1.0 at 280 nm in 10 min at 37 °C. The pepsin activity of HGJ was 36.85 U/mL, and the total proteolytic activity’ in duodenal juice was 12.35 U/mL.

The digestion was carried out in three steps: (1) “chewing” 3 min; (2) “stomach” with a gradual lowering of pH from 7–5–2.5/2 h; (3) “duodenal” pH adjusted to 7.0/1 h.

The myofibrillar or sarcoplasmic proteins were diluted up to the final protein concentration of 5% (*w*/*v*) with 0.9% NaCl solution at 37 °C. The samples were mixed in a Stomacher 400 (Seward, Norfolk, UK) for 3 min at 37 °C and a “blank” (“0”) sample was collected. Subsequently, HGJ was added at 15 U/g of protein. After mixing for 5 min in a Stomacher 400 (37 °C), the pH of the samples was reduced to 5.0 (2 M HCl) and after 10 additional min to 2.5 and the samples were then incubated for 105 min. Thereafter, pH was increased to 7.0 with a 4 M NaOH solution. At this stage, “gastric” samples (“G”) were collected. Next, HDJ was added at 31.2 U/g of protein and the digested salmon proteins extract was mixed in a Stomacher 400 (37 °C) for 60 min. “Duodenal” (“D”) samples were taken at this stage.

The samples collected at each stage of the experiment were cooled on ice and centrifuged (9000× *g*, 20 min, 4 °C). Supernatants were collected, frozen and then freeze-dried (Freezone 4.5, Labconco, Kansas City, MO, USA) and stored at −18 °C. The *ex vivo* digestion procedure was performed in triplicate.

#### 3.2.5. *In Vitro* Hydrolysis

Myofibrillar/sarcoplasmic proteins were mixed and incubated at 37 °C (Memmert 100–800 laboratory incubator, Schwabach, Germany) in Nunc-type tubes with a KL-942 laboratory rocker-shaker (JWE Electronic, Warsaw, Poland). An enzymatic pepsin preparation was used instead of HGJ in an amount corresponding to 15 U per gram of hydrolyzed protein, whereas Corolase PP was added at an amount of 31.2 U/g of hydrolyzed protein. The activity of pepsin and Corolase PP were assumed based on the manufacturer’s information. The Corolase PP solution at a concentration of 50 mg/mL contained 111.6 mg of bile salts/mL and 0.1 M NaHCO_3_. Similarly to *ex vivo* digestion, the “stomach” stage lasted 120 min, whereas the “Corolase PP” stage took 60 min at pH 7. The samples collected after each stage of *in vivo* hydrolysis were cooled in ice and centrifuged (9000× *g*, 20 min, 4 °C; Sigma 3K30 laboratory centrifuge, Osterode, Germany). Supernatants were collected, frozen and then freeze-dried (Freezone 4.5, Labconco, Kansas City, MO, USA) and stored at −18 °C. The procedure of *in vitro* hydrolysis was conducted in triplicate.

#### 3.2.6. Protein Content

The protein samples were measured with a microprotein assay according to Bradford [75], using diluted Protein Assay Dye Reagent Concentrate and BSA to determine the standard curve. Samples or BSA (0.02 mL) were mixed with Bradford Dye Reagent (1 mL, Bio-Rad, Hercules, CA, USA). The absorbance at 595 nm was measured after 5–10 min against a reagent blank (GENESYS 6, Thermo Scientific, San José, CA, USA).

The protein concentration in each sample was calculated based on a standard curve. The analyses were performed in triplicate and presented as average values.

#### 3.2.7. Sodium Dodecyl Sulfate Polyacrylamide Gel Electrophoresis (SDS-PAGE)

SDS-PAGE was carried out to evaluate the protein profile after each digestion step (“Mini-PROTEAN”, Bio-Rad, Hercules, CA, USA). The assay was performed according to standard protocols [76], using 12% and 15% separating acrylamide gels. The molecular mass markers used were the Low MW standard kit (97,000–14,400 Da, GE Healthcare Life Sciences, Little Chalfont, Buckinghamshire, UK). Staining was performed according to the standard Coomassie procedure (Bio-Rad, Hercules, CA, USA).

#### 3.2.8. Angiotensin I-Converting-Enzyme (ACE) Inhibitory Activity

ACE inhibitory activity was assayed by measuring the release of HA from the substrate HHL according to Jimsheena and Gowda [77]. The assay mixture contained 0.125 mL of a 0.05 M sodium borate buffer (pH 8.2), containing 0.3 M NaCl, 0.05 mL of 5 mM HHL and 0.025 mL of ACE (2.5 mU), which was pre-incubated with different sample concentrations. The reaction was stopped after incubation at 37 °C for 30 min by the addition of 0.2 mL of 1 M HCl. Pyridine (0.4 mL) was added followed by 0.2 mL of BSC (the order of addition of reagents is critical) and mixed by inversion for 1 min and cooled on ice. Absorbance was measured at 410 nm in a spectrophotometer (GENESYS 6, Thermo Scientific, San José, CA, USA).

The degree of ACE inhibition (%) was calculated with the following equation [78]:

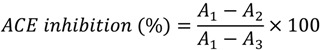
(1)
where: *A*_1_—absorbance of the ACE solution without an inhibitor (salmon protein hydrolysate); *A*_2_—absorbance of the tested sample of salmon protein hydrolysate; *A*_3_—absorbance of HHL solution (a buffer was added instead of the ACE solution and sample).

The values presented in the paper are the mean of triplicate analyses.

The IC_50_ value is defined as the concentration required to decrease the ACE activity by 50%. The percent inhibition curves were plotted using a minimum of five determinations for each sample concentration and the mean IC_50_ values were performed using GraphPad Prism^®^ v. 5.02 for Windows (GraphPad Software, La Jolla, CA, USA).

#### 3.2.9. Identification of Bioactive Peptides Using RP–HPLC–MS

The obtained protein hydrolysates were analyzed with reversed phase high performance liquid chromatography coupled with mass spectrometry (RP-HPLC–MS). A set manufactured by VARIAN^®^ (Palo Alto, CA, USA) was used in the studies; this set was composed of two pumps 212-L, a ProStar 410 autosampler, a Degassit degasser (MetaChem Technologies^®^, Torrance, CA, USA), an ESI-IT-MS-type VARIAN 500-MS mass spectrometer (with electrospraying ionization and the ion trap) and a LC/MS 12-2 nitrogen generator (Domnick Hunter Scientific^®^, Lancaster, NY, USA). Separation was performed on a Jupiter Proteo column (Phenomenex, Torrance, CA, USA) with the following parameters: 250 mm × 2 mm in size, 4 µm granule diameter and a 90 Å pore diameter that was developed to separate analytes with a low concentration of trifluoroacetic acid (TFA). 0.01% (*v*/*v*) TFA solution in water (Solution A) and in acetonitrile (Solution B) was used in a gradient of Solution B 0%–40% for 60 min. Afterwards, the column was washed and balanced with the following gradient: 40%–100% B in 60 to 65 min, 100% B in 65 to 70 min, from 100% to 0% B in 70 to 71 min and 0% B in 71 to 80 min. Separations were performed at 30 °C with 10 µL injection volume and flow velocity of 200 µL/min. The data was collected in 5–60 min. The results of analyses were stored and processed with MS WorkStation v. 6.9 software [79].

The samples of hydrolysates were dissolved in deionized water (a Synergy deionizer, Millipore^®^, Darmstadt, Germany) up to a concentration of 12.5 mg/mL of lyophilisate. The prepared solutions were centrifuged (10 min, 10,000× *g*) at room temperature. During the processing of the chromatograms, the method by Savitzky and Golay [80] that was included in the software was applied for smoothing 11 neighboring points, *i.e.*, the maximum number that may be covered by the application.

The recorded retention times were compared with the values predicted with SSRCalc software (http://hs2.proteome.ca/SSRCalc/SSRCalcX.html) [81,82,83] with a correction calculated according to the algorithm presented by Dziuba *et al.* [28] with the following equation:

*t_R predicted_* = 0.0002 × *t_R SSRCalc_*^3^ − 0.0085 × *t_R SSRCalc_*^2^ + 1.0415 × *t_R SSRCalc_* + 8.6434 (2)

ExcelTM v. 2007 software (Microsoft, Redmond, WA, USA) was used to adjust the third degree polynomial.

The molecular weights of salmon protein hydrolysis products were measured in LC-MS mode [79]. The identification of peptides was carried out with the LC-ESI-MS/MS procedure. The mass-to-charge ratio (*m*/*z*) for sought peptides and their fragment ions was determined with a Fragment Ion Calculator application that is available online (http://db.systemsbiology.net:8080/proteomicsToolkit/FragIonServlet.html) [84]. During separations, the voltages of the needle and shield were 5000 and 600 V, respectively, the pressure of spraying and drying gas was 35 and 30 psi, respectively, and the drying gas temperature was 390 °C. A single scan was averaged based on six microscans and the time of a single scan was 22.86 s. The data was collected for 60 min at 0.04 Hz. The range of the analyzed mass-to-charge ratios was 100–2000 Da.

The peptides were identified using LC-MS/MS analysis based on the mass-to-charge ratio (*m*/*z*) and the analyses of mass spectra for fragment ions derived from the precursor ion during fragmentation. The peaks that corresponded to individual ions at the same retention time allowed us to identify a given peptide [85].

The predicted retention times for the peptides with more than a four-amino acid chain were determined with Sequence Specific Retention Calculator (SSRCalc) application with a personal correction. No predicted retention times were established for di- and tripeptides as the applied application does not allow to calculate the retention times for sequences composed of two or three amino acid residues.

#### 3.2.10. Statistical Analysis

The results of the analyses are presented as means ± standard deviation. Statistical analyses were conducted with Statistica v. 10 (StatSoft, Krakow, Poland) using ANOVA Kruskal-Wallis test with a significance level of *p* < 0.05.

## 4. Conclusions

In recent years, increased attention has been paid to the biological activity of peptides of marine origin. Some ACE inhibitors that were the products of *in vitro* as well as *ex vivo* digestion of salmon proteins have become known for their anti-hypertensive bioactivity. The present study assesses the use of protein-rich salmon muscle tissue in order to exploit “hidden” ACE-inhibitory peptides. In this study, we showed that a computer-aided approach can be combined with an experimental approach for hidden ACE inhibitory peptides from salmon muscle tissue. The present results showed significant differences between peptide patterns obtained via *ex vivo* digestion and *in vitro* hydrolysis. Human gastrointestinal enzymes generated peptides with weaker ACE inhibitory activity of the hydrolysates than the activity of peptides from hydrolysates obtained with commercial porcine enzymes. Considering the complexity of digestion, the results obtained for *in vivo* digestion or, as in the present study, for *ex vivo* digestion, may differ from *in vitro* laboratory hydrolysis. Although ACE-inhibitory peptides from marine sources are weaker in ACE inhibition than synthetic drugs, the peptides derived from fish sources are often consumed in a diet. Some of the peptides found in this experiment were previously described as revealing antihypertensive activity *in vivo* in spontaneously hypertensive rats. Our results provide a basis for further clinical animal and human studies.
